# Ghrelin Regulates Glucose and Glutamate Transporters in Hypothalamic Astrocytes

**DOI:** 10.1038/srep23673

**Published:** 2016-03-30

**Authors:** Esther Fuente-Martín, Cristina García-Cáceres, Pilar Argente-Arizón, Francisca Díaz, Miriam Granado, Alejandra Freire-Regatillo, David Castro-González, María L. Ceballos, Laura M. Frago, Suzanne L. Dickson, Jesús Argente, Julie A. Chowen

**Affiliations:** 1 Hospital Infantil Universitario Niño Jesús, Department of Endocrinology, Instituto de Investigación La Princesa, University Autónoma of Madrid and Centro de Investigación Biomédica en Red (CIBER) de la Fisiopatología de la Obesidad y Nutrición (CIBEROBN), Instituto de Salud Carlos III, Madrid, Spain; 2 Instituto Cajal, Dept. of Cellular, Molecular and Developmental Neurobiology, and CIBERNED, CSIC, Madrid, Spain; 3 Dept. Physiology/Endocrine, The Sahlgrenska Academy at the University of Gothenburg, Gothenburg, Sweden

## Abstract

Hypothalamic astrocytes can respond to metabolic signals, such as leptin and insulin, to modulate adjacent neuronal circuits and systemic metabolism. Ghrelin regulates appetite, adiposity and glucose metabolism, but little is known regarding the response of astrocytes to this orexigenic hormone. We have used both *in vivo* and *in vitro* approaches to demonstrate that acylated ghrelin (acyl-ghrelin) rapidly stimulates glutamate transporter expression and glutamate uptake by astrocytes. Moreover, acyl-ghrelin rapidly reduces glucose transporter (GLUT) 2 levels and glucose uptake by these glial cells. Glutamine synthetase and lactate dehydrogenase decrease, while glycogen phosphorylase and lactate transporters increase in response to acyl-ghrelin, suggesting a change in glutamate and glucose metabolism, as well as glycogen storage by astrocytes. These effects are partially mediated through ghrelin receptor 1A (GHSR-1A) as astrocytes do not respond equally to desacyl-ghrelin, an isoform that does not activate GHSR-1A. Moreover, primary astrocyte cultures from GHSR-1A knock-out mice do not change glutamate transporter or GLUT2 levels in response to acyl-ghrelin. Our results indicate that acyl-ghrelin may mediate part of its metabolic actions through modulation of hypothalamic astrocytes and that this effect could involve astrocyte mediated changes in local glucose and glutamate metabolism that alter the signals/nutrients reaching neighboring neurons.

The coordination of energy intake and expenditure is a complex process that is influenced by both peripheral and central signals that ultimately regulate body weight and glucose homeostasis. Our understanding of the neuronal circuits controlling energy balance and metabolism has advanced considerably; however, it is only recently that glial cells have been recognized as important protagonists in this neuroendocrine process. Activation of hypothalamic microglia and astrocytes in response to high fat diet (HFD)-induced weight gain is accompanied by increased glial production of cytokines and activation of inflammatory signaling pathways in the hypothalamus^1^,^2^,^3^,^4^,^5^,^6^ which is suggested to promote central insulin/leptin resistance and metabolic disequilibrium^7^. This inflammatory process can be directly triggered by nutrients such as free fatty acids^8^, while circulating metabolic factors such as the anti-obesity hormone, leptin, can also activate glial cells^2^,^3^,^4^,^5^,^9^. Evidence has accumulated to substantiate an important role for glial cells in pathological responses to excess weight gain^1^,^2^,^3^,^4^,^9^, but their participation in the physiological control of metabolism is less well understood.

Hypothalamic astrocytes express receptors for numerous hormones involved in metabolic control, including adipostatic hormones such as leptin, but also obesity-promoting hormones such as ghrelin^6^,^10^. Leptin affects hypothalamic astrocyte morphology and their capacity to capture glucose and glutamate^4^,^5^,^9^ and the loss of leptin receptors specifically in astrocytes reduces the physiological anorexigenic response to this hormone and modifies the response to fasting and the appetite stimulating effect of ghrelin^9^, indicating the physiological importance of astrocytes in mediating this metabolic signal. However, little is known regarding the direct effect of ghrelin on astrocytes.

Ghrelin is mainly secreted by the stomach and its acylated form (acyl-ghrelin) promotes food intake primarily through activation of the growth hormone secretagogue receptor 1A (GHSR-1A)^11^. The metabolic effects of this hormone are largely opposite to those of leptin, which suppresses food intake and increases energy expenditure, due at least partially to their inverse actions on metabolic neuropeptide synthesis and secretion. Moreover, these two hormones rapidly induce opposite changes in the synaptic organization of hypothalamic metabolic circuits^12^, with astrocytes most likely participating in this reorganization^1^. Ghrelin is also involved in glucose-sensing and glucose homeostasis^13^, a process that involves hypothalamic astrocytes and their expression of glucose transporter (GLUT)2^14,15^, with aberrant hypothalamic glucose-sensing purported to be an early event in insulin resistance and type 2 diabetes^16^.

We have recently demonstrated that chronic intracerebroventricular (*icv*) administration of acyl-ghrelin to rats (i) decreases the astrocytic markers, glial fibrillary acidic protein (GFAP) and vimentin, in the hypothalamus; this occurs despite ghrelin’s effects to increase weight gain, and (ii) modulates cytokine production by astrocytes *in vitro*^10^, suggesting a direct effect of this hormone on these glial cells. Astrocytes insure correct excitatory synaptic transmission and also protect against excitotoxicity by controlling glutamate levels in the synaptic cleft^17^ and ghrelin and its mimetics have been shown to protect against glutamate excitotoxicity^18^,^19^. What remains completely unexplored is whether ghrelin has an acute effect on hypothalamic astrocyte morphology and glucose/glutamate transport.

To test the hypothesis that ghrelin modulates hypothalamic astrocyte function, we explored the effects of acute *icv* injection of acyl-ghrelin on hypothalamic astrocyte morphology and glucose and glutamate transporters. We then show that this hormone has a direct effect on glucose and glutamate uptake and metabolism in primary hypothalamic astrocyte cultures. Employing cultures of astrocytes from GHSR-1A knock-out (KO) mice and analysis of the effects of desacyl-ghrelin, an isoform that does not bind the GHSR-1A receptor, we demonstrate that at least some of the effects of acyl-ghrelin on hypothalamic astrocytes are mediated through this receptor subtype.

## Results

### Effect of acute *icv* acyl-ghrelin treatment on circulating hormone levels in male rats

There was no effect of acute acyl-ghrelin treatment on glycemia or serum insulin levels (Table 1). In control rats there was a rise in leptin levels at 24 h (F_(1,28)_ = 6.2, p < 0.02) that did not occur in acyl-ghrelin treated rats. Injection of acyl-ghrelin *icv* increased circulating levels of this hormone measured at both 1 and 24 h (F_(1,17)_ = 10.5, p < 0.005). Circulating total ghrelin levels were affected by central acyl-ghrelin administration (F_(1,26)_ = 7.6, p < 0.01) in a time-dependent manner (F_(1,26)_ = 6.6, p < 0.02), with levels being increased at 1 h, but not at 24 h (p < 0.005).

### Analysis of ghrelin receptor expression in astrocytes and cFos expression in response to *icv* acyl-ghrelin treatment

**Figure 1:**
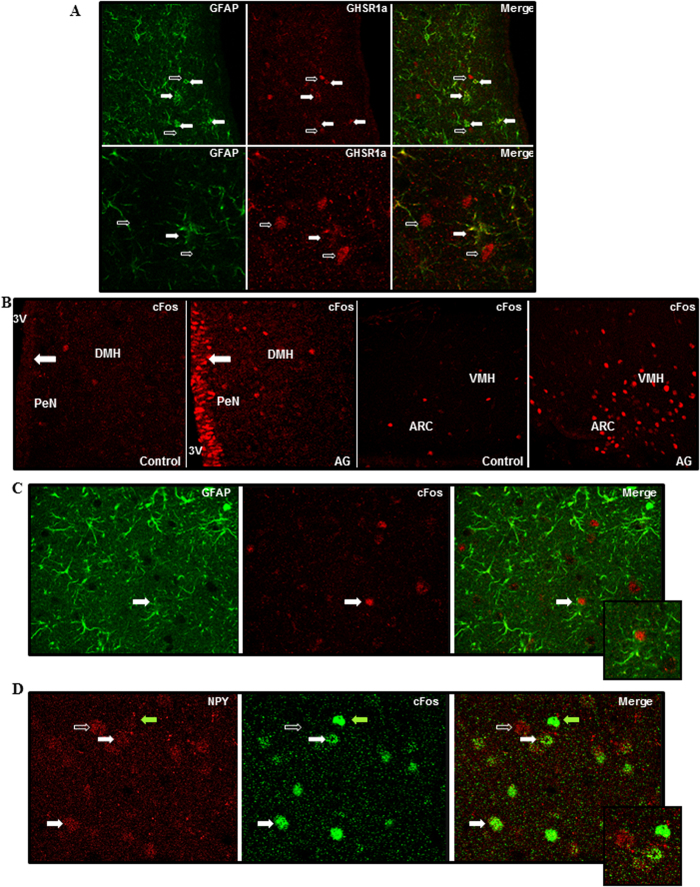
Analysis of hypothalamic glial response to intracerebroventricular acyl-ghrelin treatment. (**A**) Double immunohistochemistry for glial fibrillary acidic protein (GFAP; green) and ghrelin receptor (GHSR1a; red) in the hypothalamus. Solid arrows indicate cells positive for both GFAP and GHSR1a. Open arrows indicate cells that are immunopositive only for GHSR1a. (**B**) Immunohistochemistry for cFos in control rats and rats that were treated *icv* with acyl-ghrelin (AG) for 1 hour. There was a clear increase in cFos positive cells (red) in the hypothalamus and in cells lining the 3^rd^ ventricle (3 V) after AG treatment (solid arrow). (**C**) Double immunohistochemistry for GFAP and cFos. Solid arrow indicates a cell that is immunopositive for both GFAP and cFos. (**D**) Double immunohistochemistry for neuropeptide Y (NPY) and cFos in the arcuate nucleus. Solid errors indicate double labeled cells and the open arrow indicates a NPY neuron that is not positive for cFos. The green arrow indicates a cell only positive for cFOS. ARC = arcuate nucleus, DMH = dorsomedial hypothalamus, PeN = periventricular nucleus, VMH = Ventromedial nucleus.

Double immunohistochemistry for GFAP and GHSR-1A indicated that GFAP positive astrocytes express the ghrelin receptor and thus are capable of directly responding to this hormone (Fig. 1A). Administration of acyl-ghrelin resulted in increased expression of cFos in the hypothalamus (Fig. 1B), indicating a central response to this treatment. Cells expressing cFos were found lining the third ventricle (most likely ependymal cells or tanycytes) and dispersed throughout the hypothalamus. Although some GFAP positive cells could be found to express cFOS (Fig. 1C), the majority of cFOS positive cells were GFAP negative. In the arcuate nucleus, the majority of neuropeptide Y (NPY) immunopositive neurons also expressed cFOS (Fig. 1D).

### Effect of acute *icv* acyl-ghrelin treatment on glial structural and synaptic proteins in the male rat hypothalamus

**Figure 2:**
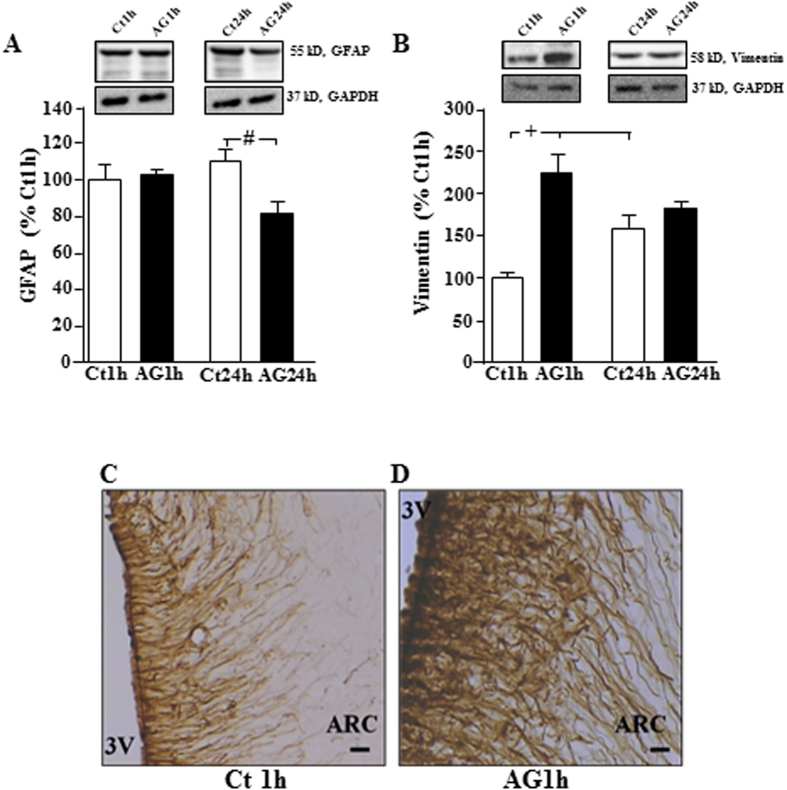
Effect of acyl-ghrelin treatment on glial structural proteins. Hypothalamic levels of (**A**) GFAP and (**B**) vimentin at 1 and at 24 hours (h) after *icv* injection of acyl-ghrelin (AG, 2 μg). Immunohistochemistry for vimentin showed increased labeling in the hypothalamus of (**C**) control and (**D**) AG treated rats. 3 V = 3^rd^ ventricle, ARC = arcuate nucleus. ^#^p < 0.01, ^+^p < 0.005. Representative Western blots are from the same gel although samples were not contiguous within the gel.

As ghrelin can rapidly induce synaptic reorganization in the hypothalamus^12^ and astrocytes modify their morphology in response to other hormones^5^,^9^ synaptic proteins and glial structural proteins and morphology were analyzed. Acyl-ghrelin had no effect at 1 h, but decreased hypothalamic GFAP levels at 24 h (F_(1,11)_ = 7.0, p < 0.03; Fig. 2A). In concordance with these results at 1 h, no modifications in the number or morphology of GFAP positive cells in the arcuate nucleus were found (data not shown).

Vimentin is expressed in tanycytes, as well as activated and immature astrocytes^20^. Levels of this structural protein were modified by acyl-ghrelin (F_(1,11)_ = 12.8, p < 0.005) in a time dependent manner (F_(1,11)_ = 5.3, p < 0.05), with a rise in vimentin at 1 h after acyl-ghrelin administration (Fig. 2B). This rise corresponded to increased labeling of projections radiating from the third ventricle that most likely represent tanycytes (Fig. 2C & D), which is in accordance with cFos activation in these specialized glial cells. In control rats, hypothalamic vimentin levels increased between 1 and 24 h, while levels in acyl-ghrelin treated rats did not change over time.

As an index of synaptic changes, the levels of proteins involved in synaptic organization and neurotransmitter release were measured. Synaptophysin (F_(1,12)_ = 5.2, p < 0.05) and syntaxin (F_(1,12)_ = 7.7, p < 0.02) levels were modified over time, with an increase between 1 h and 24 h in both control and acyl-ghrelin treated rats (Table 2). There was a time dependent effect of acyl-ghrelin on synaptotagmin (F_(1,12)_ = 5.7, p < 0.03) and SNAP25 (F_(1,12)_ = 10.0, p < 0.008), reducing synaptotagmin and increasing SNAP25 1 h after treatment. Synapsin levels were decreased with time (F_(1,12)_ = 12.4, p < 0.005) in both groups. Levels of postsynaptic density protein (PSD)95 decreased after 24 h exposure to acyl-ghrelin (F_(1,12)_ = 9.7, p < 0.009).

### Acute *icv* acyl-ghrelin treatment modifies the levels of hypothalamic glucose and glutamate transporters in male rats

**Figure 3:**
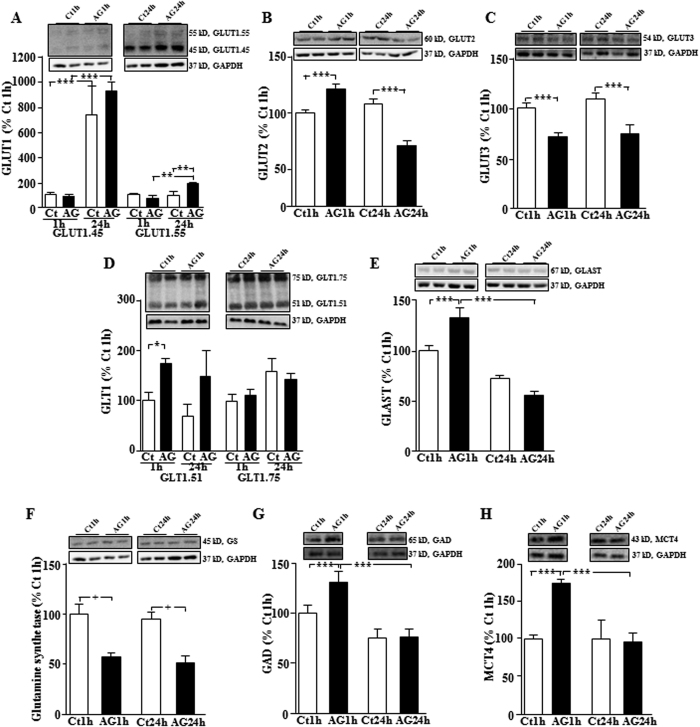
Modulation of glucose and glutamate transporters by acyl-ghrelin. Hypothalamic levels of (**A**) glucose transporter (GLUT)1, (**B**) GLUT2, (**C**) GLUT3, (**D**) glial glutamate transporter 1 (GLT1), (**E**) glutamate-aspartate transporter (GLAST), (**F**) glutamine synthetase, (**G**) glutamic acid decarboxylase (GAD), and monocarboxylate transporter (MCT)4 at 1 and at 24 hours (h) after *icv* injection of acyl-ghrelin (AG, 2 μg). *p < 0.05, ^+^p < 0.005, **p < 0.001, ***p < 0.0001. Representative Western blots are from the same gel although samples were not contiguous within the gel.

Glial cell function is substantially affected by metabolic status^4^,^5^; hence, we investigated whether acyl-ghrelin, a metabolic hormone, plays a role in this phenomenon through modification of glucose and glutamate transporters highly expressed in astrocytes. GLUT1.45, an isoform of GLUT1 expressed in most cell types in the brain including astrocytes^21^, increased over time (F_(1,11)_ = 33.1, p < 0.0001; Fig. 3A), but with no effect of acyl-ghrelin. In contrast GLUT1.55, which is expressed mainly in endothelial cells of the brain microvasculature and is responsible for the transport of glucose into the brain^22^, was modified by acyl-ghrelin in a time dependent manner (F_(1,11)_ = 10.5, p < 0.008) with no effect at 1 h, but increasing at 24 h (p < 0.01; Fig. 3A).

Levels of GLUT2, highly expressed in astrocytes and known to be involved in glucose sensing^14^,^15^, changed over time (F_(1,11)_ = 19.8, p < 0.001) in response to acyl-ghrelin treatment, increasing at 1 h and decreasing at 24 h (p < 0.001; Fig. 3B). GLUT3 is highly expressed in neurons^21^ and acyl-ghrelin decreased this glucose transporter at both 1 h and 24 h after treatment (p < 0.001; Fig. 3C).

Acyl-ghrelin differentially modulated both isoforms of the glutamate transporter GLT1, which are expressed almost exclusively in astrocytes^23^. Levels of the GLT1.51 isoform rose at 1 h (p < 0.05; Fig. 3D), with no effect at 24 h, while GLT1.75 did not change. Glutamate aspartate transporter (GLAST) levels changed over time in response to acyl-ghrelin (F_(1,11)_ = 15.1, p < 0.003), being increased at 1 h and then decreasing to control levels at 24 h (p < 0.0001; Fig. 3E).

Glutamate is metabolized to glutamine by the astrocyte-specific enzyme glutamine synthetase^24^. Levels of this enzyme were decreased by acyl-ghrelin at both 1 h and 24 h (F_(1,11)_ = 34.0, p < 0.0001; Fig 3F), suggesting a possible decrease in the levels of glutamine made available to neurons. In contrast, glutamate decarboxylase (GAD)65, which catalyzes the decarboxylation of glutamine to GABA in neurons, had a time-dependant increase in response to acyl-ghrelin (F_(1,12)_ = 16.2, p < 0.002; Fig. 3G), rising at 1 h and returning to control levels by 24 h (p < 0.003).

As the astrocyte-neuronal lactate shuttle has been implicated in the regulation of glucose homeostasis^25^, we analyzed the levels of monocarboxylate transporters (MCT) that transport both lactate and pyruvate. Acyl-ghrelin treatment did not affect MCT1, which is widely expressed in the brain including both neurons and astrocytes (Ct1h:100 ± 8.3, AG1h: 113.5 ± 11.6, Ct24h: 115.6 ± 5.5, AG24h: 93.4 ± 22.3% Ct1h) or MCT2 (Ct1h:100 ± 11.2, AG1h: 94.5 ± 28.6, Ct24h: 138.8 ± 39.0, AG24h: 190.4 ± 71.9% Ct1h). MCT4 is expressed in metabolically active cells and in the brain it is mainly found in astrocytes^26^. Levels of MCT4 increased 1 h after treatment with acyl-ghrelin and returned to basal levels at 24 h (Fig. 3H).

### Changes in hypothalamic glucose and glutamate transporters in response to chronic acyl-ghrelin treatment are seen in *ad libitum* but not pair-fed rats

**Figure 4:**
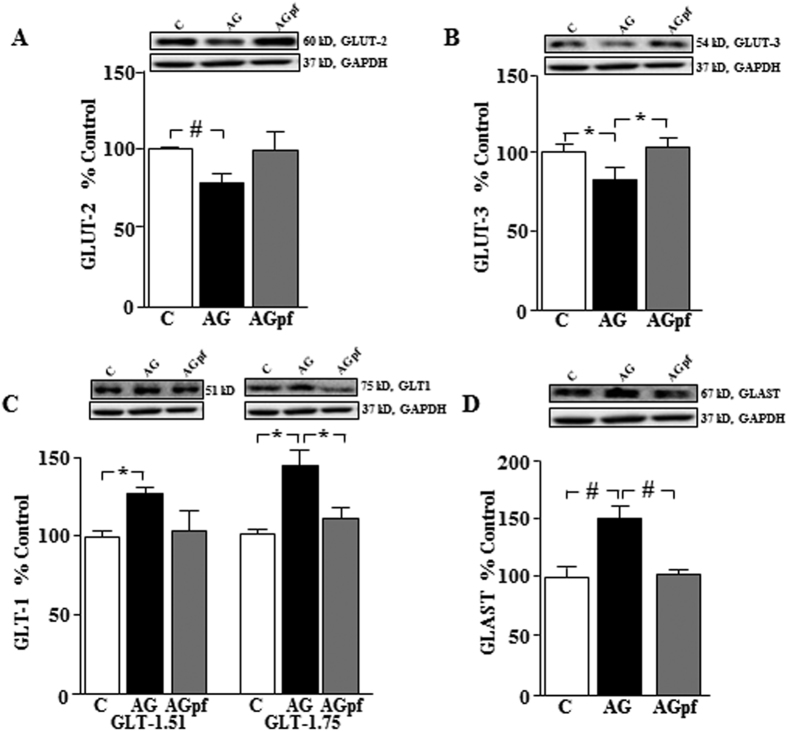
Analysis of glucose and glutamate transporters in response to chronic acyl-ghrelin treatment. Hypothalamic levels of (**A**) glucose transporter (GLUT)2, (**B**) GLUT3, (**C**) glial glutamate transporter 1 (GLT1) and (**D**) glutamate-aspartate transporter (GLAST) in male rats receiving acyl-ghrelin (AG) *icv* for 2 weeks (5 μg/day). Controls received saline and one group of AG treated rats were pair-fed (pf) with controls. *p < 0.05, ^#^p < 0.01.

As continuous exposure to increased levels of acyl-ghrelin causes metabolic changes that could be involved in the glial responses observed at 24 h, we determined how chronic exposure to increased central acyl-ghrelin affects hypothalamic glucose and glutamate transporters. This was compared to the response in acyl-ghrelin treated rats that were pair-fed to control rats in order to limit the metabolic effects of acyl-ghrelin induced food intake. There was no effect of chronic acyl-ghrelin exposure on GLUT1 levels (data not shown), while GLUT2 (p < 0.01; Fig. 4A) and GLUT3 (p < 0.05; Fig. 4B) were decreased by acyl-ghrelin in rats allowed to eat *ad libitum*. However, acyl-ghrelin had no effect on these parameters in pair-fed rats. Chronic acyl-ghrelin treatment increased both isoforms of GLT1 (p < 0.05; Fig. 4C) and GLAST (p < 0.01; Fig. 4D), but again only in *ad libitum* fed rats. Thus, the effects of long-term acyl-ghrelin treatment on glucose and glutamate transporters appear be related to the changes in metabolic parameters induced by this hormone, including weight gain, when the rats are allowed to eat *ad libitum*^10^.

### Acyl-ghrelin rapidly modifies glucose and glutamate uptake into rat hypothalamic astrocytes

**Figure 5:**
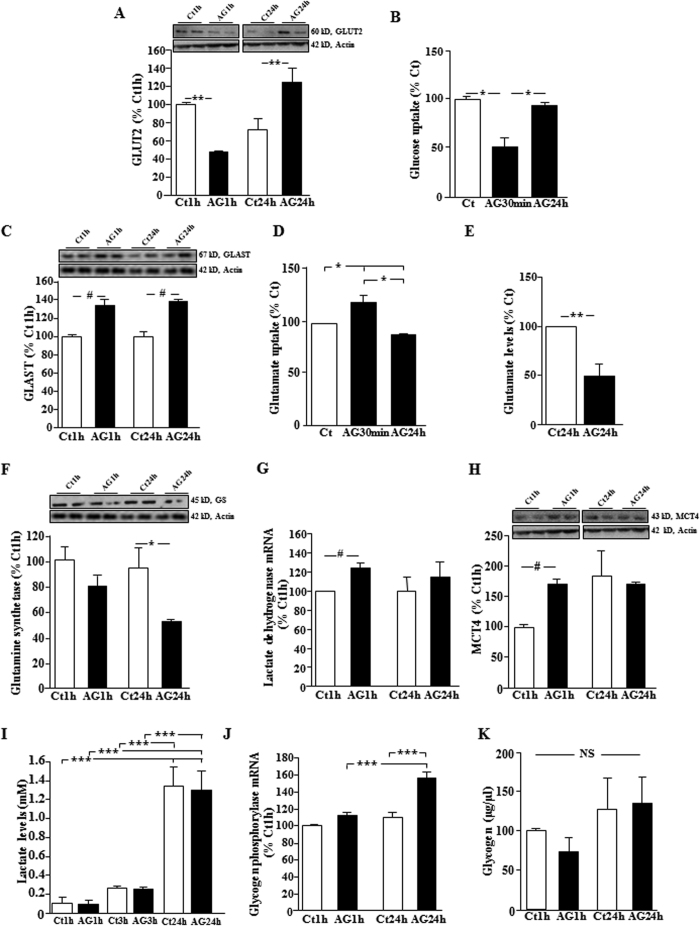
Response of hypothalamic astrocyte-enriched cultures to acyl-ghrelin treatment. Relative levels of (**A**) glucose transporter (GLUT)2, (**B**) glucose uptake, (**C**) glutamate-aspartate transporter (GLAST), and (**D**) glutamate uptake in primary astrocyte-enriched cultures from the hypothalamus of male Wistar rats treated with acyl-ghrelin (AG) or vehicle (Ct) for 1 h or 24 h. (**E**) Glutamate levels in the media of primary astrocyte cultures decreased after 24 h exposure to AG. Relative levels of (**F**) glutamine synthetase, (**G**) lactate dehydrogenase, (**H**) monocarboxylate transporter (MCT)4, (**J**) glycogen phosphorylase and (**K**) intracellular glycogen content were measured in primary astrocyte-enriched cultures from the hypothalamus of male Wistar rats treated with acyl-ghrelin (AG) or vehicle (Ct) for 1 h or 24 h. Lactate levels (**I**) were measured in the media of primary astrocyte cultures after 1, 3 or 24 h exposure to AG. *p < 0.05, ^#^p < 0.01, **p < 0.001, ***p < 0.0001, NS = not significant. Representative Western blots are from the same gel although samples were not always contiguous within the gel.

Leptin has time-dependent effects on GLUT2 and GLAST levels and glucose and glutamate uptake into hypothalamic astrocytes *in vitro*^4^; therefore, we analyzed these parameters after acyl-ghrelin treatment for 1 h and 24 h. Total GLUT2 levels were decreased at 1 h and increased at 24 h (p < 0.001; Fig. 5A). This is consistent with the observation that acyl-ghrelin treatment rapidly decreased glucose uptake in primary astrocyte cultures that increased to control levels with 24 h exposure (p < 0.05; Fig. 5B). GLUT1 and GLUT3 levels were unaffected by acyl-ghrelin in astrocyte cultures (data not shown). As glucokinase is important for central glucose-sensing^27^ and this enzyme is expressed in glial cells^28^, we determine whether acyl-ghrelin modulated the expression of this enzyme in hypothalamic astrocytes. No significant effect of AG was found (Ct1h:100 ± 15.3, AG1h: 103.3 ± 21.9, Ct24h: 144.6 ± 40.0, AG24h: 130.6 ± 22.0% Ct1h).

GLAST levels were increased in astrocytes *in vitro* at both 1 h and 24 h after exposure to acyl-ghrelin (p < 0.01; Fig. 5C), with no significant effect on GLT1 levels (data not shown). Acyl-ghrelin exposure resulted in rapid glutamate uptake into astrocytes (p < 0.05; Fig. 5D) that then decreased after 24 h of treatment. Glutamate concentrations decreased in the culture media at 24 h after acyl-ghrelin treatment (p < 0.001; Fig. 5E), with no significant effect seen at 1 h or 2 h (data not shown).

Although glutamate uptake into astrocytes rose, glutamine synthetase levels, which catalyzes the conversion of glutamate to glutamine^24^, were unchanged at 1 h and decreased at 24 h after treatment (p < 0.05; Fig. 5F). This suggests that although acyl-ghrelin stimulates astrocytes to rapidly take-up glutamate, this process may not be accompanied by increased conversion of glutamate to glutamine. The glutamate-glutamine cycle is a physiological process in situations of increased glutamate neurotransmission whereby astrocytes produce glutamine that is then supplied to neurons in order for these cells to replenish their glutamate stores^29^. Glutamate uptake into astrocytes also stimulates lactate production, at least *in vitro*^30^, and we found that the acyl-ghrelin stimulated increase in glutamate uptake was associated with increased expression of lactate dehydrogenase at 1 h (p < 0.003; Fig. 5G). This was associated with a rise in MCT4 levels at 1 h (p < 0.001; Fig. 5H), suggesting a rapid increase in lactate transport by astrocytes. However, lactate levels in the culture media did not change in response to acyl-ghrelin treatment, but increased with time (p < 0.0005; Fig. 5I). Protein levels of MCT1 were undetectable by Western blotting.

Astrocytes store glycogen that can be mobilized in situations of energy deficit^31^. The mRNA levels of glycogen phosphorylase, the rate limiting enzyme in glycogenolysis^32^, were stimulated by acyl-ghrelin (p < 0.0003) in a time dependant manner (p < 0.01), reaching significance at 24 h (Fig. 5J). Although there was a tendency to decrease in response to acyl-ghrelin at 1 h, the cellular glycogen levels in astrocytes did not change significantly (Fig. 5K). Insulin stimulated glycogen storage in astrocytes (154% of control levels at 24 h), as previously reported^33^.

### Acyl-ghrelin modifies glucose and glutamate transporters in astrocytes from mice through the GHSR-1A receptor

**Figure 6:**
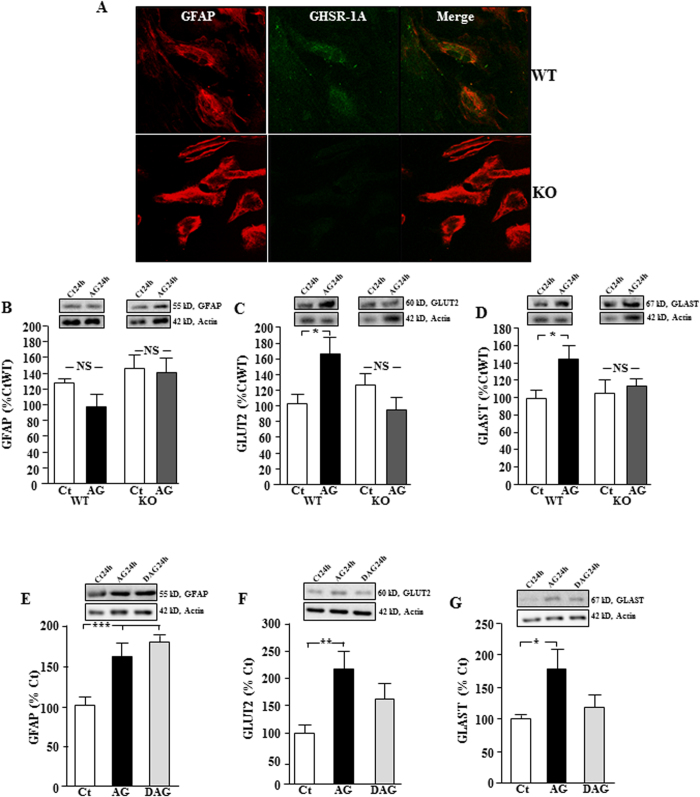
Role of GHSR-1A in the response of astrocytes to ghrelin. Double immunofluorescence for GHSR-1A (green) and glial fibrillary acidic protein (GFAP, red) in cultures of hypothalamic astrocytes of wild-type (WT) and GHSR-1A (knock-out, KO) male mice (**A**). Levels of (**B**) GFAP, (**C**) glucose transporter (GLUT)2 and (**D**) glutamate-aspartate transporter (GLAST) in primary hypothalamic astrocyte-enriched cultures of WT and GHSR-1A KO male mice treated with acyl-ghrelin (AG) or vehicle (Ct). In order to further analyze the involvement of GHSR-1A in the response of astrocytes to acyl-ghrelin (AG), primary hypothalamic astrocyte-enriched cultures were treated with AG, desacyl-ghrelin (DAG) or vehicle (Ct) and the relative levels of (**E**) GFAP, (**F**) GLUT2 and (**G**) GLAST were measured. *p < 0.05, **p < 0.001, ***p < 0.0001, NS = not significant. Representative Western blots are from the same gel although samples were not always contiguous within the gel.

To determine whether the direct effect of acyl-ghrelin on astrocytes is mediated through GHSR-1A, primary cultures from WT and GHSR-1A KO mice were employed. Immunoreactive GHSR-1A could be detected in WT cultures, but not in those from KO mice (Fig. 6A).There was no effect of acyl-ghrelin on GFAP levels in cultures from WT or GHSR-1A KO mice (Fig. 6B). In contrast, acyl-ghrelin increased GLUT2 (p < 0.05; Fig. 6C) and GLAST (p < 0.05; Fig. 6D) in WT cultures, but had no effect in astrocytes cultured from GHSR-1A KO mice, suggesting that this effect is mediated through GHSR-1A. Although there was no effect of acyl-ghrelin on GFAP in astrocyte cultures from WT or GHSR-1A KO mice, we have previously reported that this hormone stimulates GFAP in astrocytes from rats^10^, indicating a possible difference between species.

We next treated rat hypothalamic astrocyte cultures with acyl-ghrelin or desacyl-ghrelin to determine whether the stimulatory effect on GFAP and glucose and glutamate transporters in rats is specific to acyl-ghrelin. Both ghrelin isoforms stimulated GFAP levels in these cultures (p < 0.0001; Fig. 6E), suggesting that this response is not mediated, at least exclusively, through GHSR-1A in rat astrocytes. In contrast, GLUT2 (p < 0.005; Fig. 6F) and GLAST (p < 0.05; Fig. 6G) were increased by acyl-ghrelin but not by desacyl-ghrelin, corroborating the results in the KO mice indicating that this effect is likely mediated through GHSR-1A.

## Discussion

It is becoming increasingly clear that astrocytes have a multifactorial participation in the neuroendocrine control of metabolism, with these glial cells having both physiological and pathophysiological actions^4^,^34^,^35^. Here we show that the orexigenic hormone acyl-ghrelin can rapidly modulate hypothalamic levels of glucose and glutamate transporters highly expressed in astrocytes, both *in vitro* and *in vivo*, and the ability of these glial cells to transport glucose and glutamate *in vitro*. This suggests some effects of this hormone on metabolic circuits could be mediated through astrocytic modification of local glucose and glutamate levels, which could in turn affect neuronal excitability and activity, as well as survival.

Glucose is the main fuel source for the brain and astrocytes play an important role in its uptake and transport within the brain^36^. Glucose is transported into the CNS through GLUT1 located in the endfeet of astrocytes surrounding capillaries^37^, but acyl-ghrelin did not modify the astrocytic isoform of this transporter *in vivo* or *in vitro*, although it would be of interest to determine levels specifically in the endfeet of these glial cells. In neurons, glucose uptake is primarily mediated through GLUT3^38^ and acyl-ghrelin rapidly decreased hypothalamic levels of this transporter. Hypoglycemia, which stimulates ghrelin secretion^39^, also reduces hypothalamic GLUT3 expression^40^. Thus, acyl-ghrelin could be involved in signaling a reduction in glucose levels to the hypothalamus in situations of negative energy balance such as fasting, inducing a switch to alternative energy sources.

Astrocytes generate lactate that can then be used by neighboring neurons as an alternative energy source and this is especially important in situations of low glucose availability^36^. An acyl-ghrelin induced rise in lactate availability to neurons is suggested by its stimulation of lactate dehydrogenase and MCT4 levels both *in vivo* and *in vitro*. Moreover, an increase in glycogen phosphorylase indicates that acyl-ghrelin stimulates the hydrolysis of glycogen stores in astrocytes, which can contribute to a rise in lactate production, which could also result from the rise in glutamate uptake^36^; however, we saw no significant change in glycogen levels suggesting that they may be rapidly restored. Although the rise in MCT4 levels suggests an increase in lactate transport, we detected no effect of acyl-ghrelin on extracellular lactate concentrations. Lactate levels increased dramatically overtime in the media from all cultures; however, as the concentration gradient in the *in vitro* system favored the outward flow of lactate^41^, the possible contribution of ghrelin to increased lactate secretion could have been masked. Lactate can also be metabolized back to pyruvate, which is also transported by MCT4. Although more studies are clearly necessary to determine how acyl-ghrelin directly modifies astrocyte metabolism and transport of nutrients to neurons, these cells do respond to this orexigenic signal. Hypothalamic GLUT3 levels were decreased at 24 h after acute acyl-ghrelin treatment and with chronic *icv* acyl-ghrelin treatment. However, this long-term suppression may not be a direct effect of acyl-ghrelin, as GLUT3 returns to control levels when rats are pair-fed even though circulating acyl-ghrelin levels remain high in these animals^10^. We previously demonstrated that hypothalamic GLUT3 levels decrease with chronic *icv* leptin treatment and in response to leptin in a hypothalamic neuronal cell line^4^. Circulating leptin levels are increased in acyl-ghrelin treated rats, but not when they are pair-fed^10^, suggesting that leptin could mediate this long-term effect on GLUT3. Moreover, after 24 h of acyl-ghrelin treatment, GLUT3 levels are unaffected in this hypothalamic neuronal cell line (unpublished personal data), further suggesting that the long-term effect of acyl-ghrelin on GLUT3 levels in hypothalamic neurons is indirect.

Astrocytes in brain areas involved in the control of food intake express high levels of GLUT2^42^,^43^. Hypothalamic astrocytes participate in glucose sensing, with their expression of GLUT2 being essential for this process^42^,^43^,^44^. As ghrelin affects systemic glucose equilibrium through actions at the hypothalamic level^11^,^45^ and given the results presented here, one might speculate that direct acyl-ghrelin modulation of astrocyte GLUT2 levels and glucose transport could impact on the glucose-sensing mechanism. In astrocyte cultures acyl-ghrelin rapidly decreased GLUT2 levels and this was associated with decreased glucose uptake. These parameters increased or returned to control levels by 24 h of exposure. This time-dependent effect could indicate the more acute effects of ghrelin as observed in the physiological pre-prandial rise in its circulating levels versus prolonged hyperghrelinemia, as in fasting or starvation^11^,^46^. The preprandial rise in ghrelin is coincident with increased glycemia and a decrease in insulin before food intake commences^47^. A rapid decline in GLUT2 could decrease the glucose-sensing ability of astrocytes^42^ that would increase ingestion, which is one function of ghrelin^11^. Increased ghrelin during starvation is involved in maintaining glucose levels^11^. However, the *in vitro* effects of acyl-ghrelin on GLUT2 levels in astrocyte cultures were opposite to those seen *in vivo*. GLUT2 could be regulated differently in other cell types, such as neurons, or other glial cells that have been shown to respond to ghrelin, including microglia^48^ and oligodendrocytes^49^. Moreover, indirect effects of acyl-ghrelin on hypothalamic astrocytes, such as stimulation of gliotransmitter release, have also been described^35^,^50^. Energy transport and metabolism are tightly coupled between astrocytes and neurons^36^ and this cross-talk is lacking in the *in vitro* astrocyte system, thus modifying the normal temporal changes in inputs received by these glial cells.

Tanycytes also express GLUT2^44^ and appear to respond rapidly to acyl-ghrelin *in vivo*, as we found cFos expression in cells lining the 3 V and vimentin labeled projections from the 3 V were increased with no indication of vimentin positive astrocytes being found. Tanycytes occupy a strategic location for the transport of metabolic factors to the hypothalamus^51^, are a source of progenitor cells for the regeneration of hypothalamic metabolic circuits^52^ and are involved in glucose sensing^44^. Whether ghrelin mediates any of these metabolic functions of tanycytes deserves future investigation, but it is of note that tanycytes were recently demonstrated to transport both acyl-ghrelin^53^ and desacyl-ghrelin^54^ across the blood-brain barrier, similar to what has also been reported for leptin entry into the brain^55^. Thus, these specialized cells appear to play an important regulatory role in the transport of metabolic hormones. Moreover, a decreased ability of tanycytes to transport ghrelin could possibly contribute to some situations of increased weight gain^53^.

Vimentin increased between 1 and 24 hours in control rats. Hypothalamic levels of this structural protein are modulated by melatonin^56^, glucocorticoids^57^ and thyroid hormone^58^. Levels of these hormones fluctuate throughout the day; hence, it is possible that hypothalamic vimentin levels are physiologically modulated in a diurnal or circadian fashion. However, this possibility, as well as whether AG participates in generating a rhythmic expression of vimentin, remains to be determined.

Acyl-ghrelin also stimulated rapid glutamate uptake by astrocytes *in vitro*. Glutamate transport by astrocytes not only modulates synaptic transmission and produces neuroprotection^17^, but it also activates glycolysis in these cells, increasing both the production and release of lactate to be used as an alternative fuel source for neurons during increased activation^36^. This is in concordance with the acyl-ghrelin induced increase in the expression of MCT4 and lactate dehydrogenase, as discussed above. Glutamate transporters are highly expressed on astrocytes, with GLT1 being found almost exclusively in astrocytes and GLAST in astrocytes and other glial cells^23^. Both GLT1 and GLAST were increased rapidly by acyl-ghrelin *in vivo*, but *in vitro* this effect was only seen on GLAST. These two glutamate transporter are involved in different functions, such as neurotransmission versus neuroprotection, within the same tissue^59^ and thus, the mechanisms that control their expression may also differ.

Ghrelin potentiates glutamate release to activate NPY/AgRP neurons^60^ and, as shown here, also rapidly stimulates the uptake of glutamate by astrocytes, which would function to avoid excess excitability and excitotoxicity to neighbouring neurons. Indeed, ghrelin and it mimetics to protect against glutamate excitotoxicity^18^,^19^.

Within astrocytes, glutamate can be metabolized to glutamine via glutamine synthetase or be shuttled into the tricarboxylic acid cycle (TCA). Glutamine released by astrocytes can be taken-up by neurons where it can be reconverted to glutamate via glutaminase enzymatic actions. This astrocyte-mediated glutamate recycling is coupled to glucose glycolysis where the resulting lactate is then released for neuronal uptake and oxidation^61^. Although glutamate uptake is stimulated by acyl-ghrelin, glutamine synthetase levels were decreased both *in vivo* and *in vitro*, suggesting that glutamate could be shuttled into the TCA cycle. Indeed, when external glutamate levels are low the glutamine synthetase pathway is favored, but when glutamate concentrations are high, oxidative processes are recruited, with considerable glutamate being consumed via astrocytic malic enzyme^62^. Glutamate consumption in the brain also increases in hypoglycemic states^63^ when ghrelin levels would be increased. Acute *icv* acyl-ghrelin administration also stimulated GAD levels, suggesting increased GABA production. Indeed, ghrelin stimulates GABA release in the hypothalamic through astrocyte release of ATP^50^. Likewise, fasting increases hypothalamic production of GABA, possibly through increased ghrelin secretion^64^. Together these results suggest that astrocytes participate in acyl-ghrelin’s effects on hypothalamic synaptic transmission, and possibly neuronal metabolism.

Rapid modifications in synaptic inputs in the arcuate nucleus occur in response to ghrelin^12^. Here we found synaptotagmin-1 to be decreased and SNAP25 to be increased within one hour after *icv* acyl-ghrelin injection, with both returning to control levels at 24 h. Synaptotagmin-1 acts as a calcium-sensor in Ca^2+^-dependent release of neurotransmitter vesicles and is important for the local functioning of GABAergic/glutamatergic reciprocal connections^65^. The rapid increase in the membrane SNARE protein SNAP25 could indicate an increase in secretory vesicle turn-over. As PSD95 is an integral postsynaptic component of glutamatergic synapses, a reduction in this protein after 24 h of exposure to acyl-ghrelin suggests a reduction in excitatory inputs in the hypothalamus, similar to what was reported by Pinto and colleagues^12^.

The effects of acyl-ghrelin are mediated at least in part through GHSR-1A, as acyl-ghrelin did not modulate GLUT2 or GLAST levels in astrocyte cultures from mice lacking this receptor. Moreover, deacyl-ghrelin did not induce these effects in rat hypothalamic astrocytes. In contrast, both acyl- and deacyl-ghrelin stimulated GFAP levels *in vitro*, indicating a GHSR-1A independent effect. Glucose and glutamate transport in response to acyl-ghrelin were not measured in mouse astrocytes. Although one might expect similar responses as those observed in rats, given that changes in transporters were similar, this has not been demonstrated.

The results reported here add to the expanding functions attributed to astrocytes in the neuroendocrine control of metabolism. Rapid changes in astrocyte functions in response to metabolic hormones appear to play an important role in the normal physiology of metabolic circuits. Astrocytic responses change with prolonged exposure to metabolic hormones, indicating that they could also mediate some of the pathophysiological effects of prolonged exposure to these substances. Thus, in order to completely understand how metabolic circuits work and to target them for treatment of conditions such as obesity, the functions of glial cells must also be taken into consideration.

## Materials and Methods

### Ethical statement

All experiments were designed according to the European Union and local laws (Royal decree: 53/2013, EU Council Directive: 2010/63/EU) for animal care. The studies were approved by the appropriate local institutional ethical committees (The Scientific Committee of the Hospital Infantil Universitario Niño Jesús, The Committee for Animal Welfare of the Universidad Autónoma de Madrid, and The Ethics Committee of the University of Gothenburg).

### Acute icv acyl-ghrelin treatment of adult male rats

Adult male Wistar rats (250–300 g; n = 16/group; Harlan InterfaunaIbérica S.A., Barcelona, Spain) were allowed to acclimate for 1 week before surgeries were performed. Rats were anesthetized (0.02 ml ketamine per 100 g of body weight (bw) and 0.04 ml xylacine per 100 g bw) and a cannula attached to a catheter was implanted in the left lateral cerebral ventricle (0.08 mm anteroposterior, 0.16 mm lateral from Bregma). Twenty-four hours after surgery, rats received either acyl-ghrelin (2 μg in 5 μl; ANASPEC, Fremont, CA, USA) or saline (5 μl; Ct). Rats were killed by decapitation at either 1 h or 24 h after the injection. The brains were removed and rapidly frozen on dry ice and stored at −80 °C. Trunk blood was collected, allowed to clot, centrifuged and the serum collected and stored at −80 °C.

To determine the acute effects of ghrelin on astrocyte morphology and cellular activation in response to treatment, acyl-ghrelin or saline treated rats (n = 5) were perfused transcardially (4% paraformaldehyde, pH 7.4) under pentobarbital anesthesia (1 mg/kg) 1 h after treatment. The brains were removed, post-fixed in the same fixative overnight at 4 °C and stored in cryoprotection solution (30% sucrose, 30% ethylene glycol, in PB) at −20 °C until processed.

### Chronic icv acyl-ghrelin treatment

To compare the rapid effects with the long-term effects of acyl-ghrelin on hypothalamic glutamate and glucose transporters, our study included rats that were treated *icv* with acyl-ghrelin for two weeks. The metabolic parameters in these rats have been reported previously^10^.

Briefly, adult male Wistar rats were treated as in the acute injection study except that a minipump (Alzet. Durect Co, Canada) that delivered either 5 μg/day (0.208 μg/hour) acyl-ghrelin or saline for 14 days was connected to the cannula that was implanted in the left lateral ventricle. All rats were given free access to food and water except the pair-fed acyl-ghrelin-treated group that received the same amount of food that the controls had consumed the previous day.

Correct placement of the cannula was confirmed when the brains were processed.

### Primary astrocyte cultures from Wistar rats

Primary astrocyte cultures of the hypothalami from two-day old male Wistar rats were prepared as previously described^4^,^10^. Cultures were serum starved for 24 h and then treated with 100 nM of recombinant rat acyl-ghrelin or vehicle for 1, 3 or 24 h. Each treatment was done in triplicate in each experiment and each experiment repeated 4–6 times (n = 4–6).

To determine the specificity of the response to acyl-ghrelin and to determine if this hormone was acting through GHSR-1A, in a set of experiments cultures were treated with vehicle, acyl-ghrelin (100 nM) or desacylated (desacyl)-ghrelin (100 nM), which does not bind to GHSR-1A^11^, for 24 h.

### Primary astrocyte cultures from WT and GHSR-1A KO mice

Hypothalami were removed from two-day-old wild-type or KO male littermates from GHSR-1A KO mice^66^ and processed as above except that each hypothalamus was seeded separately to allow for the posterior determination of genotype. For immunocytochemistry, a sterilized round coverslip was placed in the culture wells before plating the cells. These cells were then processed for immunolabeling as stated below.

Dose response curves were performed to determine the appropriate dose of acyl-ghrelin to be used. The lowest dose with the maximum effect was selected.

### Protein extraction and Western blotting

The hypothalamic and primary astrocyte cultures were processed and protein concentration determined as previously reported^4^. Western blotting was performed as previously described^4^,^5^. The sources of the primary antibodies employed were as follows: GFAP (cat.# G9269) and vimentin (cat.# V6389) from Sigma, GLUT1 (cat.# GT12-A), GLUT2 (cat.# GT21-A), GLUT3 (cat.# GT31-A) and GLAST (cat.# GLAST11-A) from Alpha Diagnostic International (San Antonio, TX), GLT1 (cat.# PA3-040A) from Affinity BioReagents (Golden, CO), GAD65/67 (cat.# AB1511), MCT1 (cat.# AB3550) and MCT4 (AB3314P) from Millipore, glutamine synthetase (cat.# G7121) from USBiological (Swampscott, MA), synaptophysin (cat.# RM-9111S0) from ThermoScientific (Rockford, IL) and synapsin I (cat.# 574778), synaptotagmin (cat.# 573826), syntaxin (cat.# 574784), SNAP25 (cat.# 487912) and PSD95 (cat.# 529541) from Calbiochem (San Diego, CA). Anti-MCT2 (cat.# sc-50323) and actin (cat.# sc-1616) from Santa Cruz Biotechnology (Santa Cruz, CA) and anti-glyceraldehyde 3-phosphate dehydrogenase [GAPDH (cat.# 54593) from ANASPEC] were used to normalize sample loading variability. Secondary antibodies conjugated with peroxidase were from Pierce Biotechnology (Rockford, IL). Bound peroxidase activity was visualized by chemiluminescence and quantified by densitometry using a Kodak Gel Logic 1500 Image Analysis system and Molecular Imaging Software, version 4.0 (Rochester, NY). All data are normalized to control values on each gel.

### Immunohistochemistry

Coronal brain sections (40 μm) were cut on a vibratome and stored in cryoprotection solution at −20 °C. For immunohistochemistry, brain sections containing the hypothalamic arcuate nucleus were washed in PB, incubated in 50% methanol containing 3% hydrogen peroxide for 20 min and then washed twice for 20 min in 0.1 M PB with 0.3% bovine serum albumin and 0.3% Triton X-100. This buffer was also used in the subsequent washes and incubations. Free-floating sections were incubated overnight at 4 °C with anti-GFAP or anti-vimentin at a dilution of 1:500, washed twice and incubated for 2 hr with horseradish peroxidase conjugated goat anti-mouse IgG (Pierce Biotechnology, 1:500) at RT. Peroxidase activity was revealed with 0.01% hydrogen peroxide, using 3,3′-diaminobenzidine as the chromogen. Immunostaining was absent when the primary antibody was omitted. All sections from both groups were assayed in parallel.

For immunofluorescent staining, tissue sections were rinsed in PB and then incubated in the same buffer as above. The free-floating sections were incubated 72 h with anti-GFAP (1:500), anti-GHSR1 (cat.# sc-10362 Santa Cruz Biotechnology, 1:200), anti-cFOS (cat.# sc-52; Santa Cruz Biotechnology, 1:500), anti-NPY (cat.# ABS 028-08-02 ThermoFisher, 1:500) or a combination of these antibodies. Astrocyte cultures were first fixed with paraformaldehyde (4%) and rinsed in PB. The antibodies (anti-GFAP and anti-GHSR1) and washes were added directly in the culture wells. For GFAP detection, sections were incubated with anti-mouse Alexa Fluor 488 (1:1000, Molecular Probes). For GHSR1 and cFOS detection, sections were incubated with biotin labeled anti-goat or anti-rabbit IgG (1:1000, Thermo Scientific), respectively, for 2 h followed by incubation with streptavidin Alexa Fluor 633 (1:1000, Molecular Probes) for 2 h. Immunofluorescence was visualized with a scanning confocal microscope. The specificity of the GHSR1 antibody was previously tested^67^ with these authors showing no labeling by this antibody in GHSR1 KO mice.

### Analysis of glial cell morphology

Six sections/rat, distributed from −2.3 to −3.3 mm from Bregma containing the arcuate nucleus were analyzed. Images from fifteen rectangular fields of the arcuate nucleus corresponding to an area of 19.5 mm^2^ in each section were captured at 40X by using a digital camera and Image-Pro Plus software (Media Cybernetics Inc, Silver Spring, MD). The number of GFAP+ cells per field, the number of projections per cell and the mean projection length were determined by using ImageJ. All morphometric analyses were performed without previous knowledge of the experimental group from which the sections were obtained.

### Real time-PCR

Extraction of total RNA and real time-PCR were carried out in hypothalamic astrocyte cultures as previously described^5^. Predesigned primers from Applied Biotechnology were used to measure relative expression levels of lactate dehydrogenase (ldha, Rn00820751), glucokinase (Rn00561265) and glycogen phosphorylase (pygb, Rn01536623) and 18 s (rps18, Rn01428915) was used as the internal control.

### Glycogen content in astrocyte cultures

Cellular glycogen content in response to acyl-ghrelin was measured in hypothalamic astrocytes according to the manufacturer’s instructions (Sigma). Insulin treatment (50 nM) was used as a positive control.

### Glutamate and lactate concentrations in cell culture media

After incubation of primary hypothalamic astrocyte cultures with acyl-ghrelin or vehicle for 1, 3 or 24 h as described above, 1 ml of media was removed from each culture and lyophilized (n = 3 independent experiments). Glutamate (BioVision, Mountain View, CA, USA) and lactate (EnsyChrom, BioAssay Systems, Hayward, CA, USA) determinations were performed following the manufacturers’ instructions.

### Glucose uptake by astrocytes

^8,36^-2-deoxy-glucose ([^3^H]-2-DG) uptake experiments were conducted as previously described^4^. Astrocytes (+ were either treated with acyl-ghrelin (100 nM) for 24 h or added for 10 min during the preincubation period and maintained during the incubation. Blanks, which were similarly processed but incubated at 0 °C, accounted for 20% of the radioactivity in control cultures and this was subtracted from all measurements. All experiments were performed in triplicate and using primary astrocyte cultures from at least 5 different male pups.

### Glutamate uptake by astrocytes

Glutamate uptake was performed as previously described^4^. Blanks, which were similarly processed but incubated at 0 °C, accounted for 20% of the radioactivity in control cultures and this was subtracted from all measurements. All experiments were performed in triplicate and using primary astrocyte cultures from at least 5 different male pups.

### Statistical analysis

All data are presented as mean ± SEM. A two-tailed Student’s *t* test was used for comparison between 2 groups. Two-way ANOVA was used to determine the effect of acyl-ghrelin over time. Scheffe’s F test was used for posthoc analysis. The values were considered significantly different when the p value was lower than 0.05. All Western blotting results are reported as the percent of the control value. In *in vitro* studies the n represents the number of individual experiments performed.

## Additional Information

**How to cite this article**: Fuente-Martín, E. *et al*. Ghrelin Regulates Glucose and Glutamate Transporters in Hypothalamic Astrocytes. *Sci. Rep*. **6**, 23673; doi: 10.1038/srep23673 (2016).

## Figures and Tables

**Table 1:** Glycemia and hormone levels after acylated ghrelin treatment.

	Ct1h	AG1h	Ct24h	AG24h	ANOVA
Glycemia (mg/dl)	97.4 ± 2.5	90.5 ± 3.3	94.6 ± 3.4	89.1 ± 3.1	NS
Insulin (ng/ml)	3.2 ± 0.6	3.3 ± 0.3	2.4 ± 0.5	2.3 ± 0.6	NS
Leptin (ng/ml)	4.2 ± 0.5	3.7 ± 0.2	6.2 ± 0.9^a^	3.7 ± 0.6^b^	P < 0.03
AG (pg/ml)	171.0 ± 14.2	245.3 ± 17.4^a^	176.1 ± 27.4	244.2 ± 18.9^b^	P < 0.04
Total ghrelin (pg/ml)	1173.4 ± 89.1	3176.9 ± 594.6^a^	1007.6 ± 75.2	1355.5 ± 256.6^c^	P < 0.002

Glycemia and circulating hormone levels in response to *icv* treatment with acyl-ghrelin (AG) after 1 h and 24 h.

^a^Different from control (Ct)1h.

^b^Different from Ct24h.

^c^Different from AG1h.

**Table 2:** Changes in synapto-proteins in response to acylated ghrelin.

	Ct1h	AG1h	Ct24h	AG24h	ANOVA
Synaptophysin	100 ± 6.1	78.2 ± 8.3	120.5 ± 7.7	104.4 ± 16.1	Time: p < 0.05
Syntaxin	100 ± 5.9	81.4 ± 4.8	122.5 ± 10.9^a^	113.3 ± 14.5^c^	P < 0.05
Synaptotagmin	100 ± 16.0	64.6 ± 8.9^a^	77.5 ± 12.8	95.8 ± 3.2^c^	P < 0.03
SNAP25	100 ± 7.7	137.8 ± 6.3^a^	127.5 ± 16.9	91.7 ± 12.5^c^	P < 0.008
Synapsin 1	100 ± 4.1	77.3 ± 3.6	54.1 ± 6.3^a^	54.5 ± 6.9^c^	P < 0.005
PSD95	100 ± 10.7	94.7 ± 18.0	85.5 ± 4.4	64.2 ± 21.1b,^c^	p < 0.009

Changes in hypothalamic synapto-protein levels in response to *icv* treatment with acylated ghrelin (AG) after 1 h and after 24 h.

^a^Different from control (Ct)1h.

^b^Different from Ct24h.

^c^Different from AG1h.

## References

[b1] Horvath T. L. . Synaptic input organization of the melanocortin system predicts diet-induced hypothalamic reactive gliosis and obesity. Proc Natl Acad Sci USA 107, 14875–14880, doi: 10.1073/pnas.1004282107 (2010). 20679202 PMC2930476

[b2] Thaler J. P. . Obesity is associated with hypothalamic injury in rodents and humans. J Clin Invest 122, 153–162, doi: 10.1172/JCI59660 (2012). 22201683 PMC3248304

[b3] Gao Y. . Hormones and diet, but not body weight, control hypothalamic microglial activity. Glia 62, 17–25, doi: 10.1002/glia.22580 (2014). 24166765 PMC4213950

[b4] Fuente-Martin E. . Leptin regulates glutamate and glucose transporters in hypothalamic astrocytes. J Clin Invest 122, 3900–3913, doi: 10.1172/JCI64102 (2012). 23064363 PMC3484452

[b5] Garcia-Caceres C. . Differential acute and chronic effects of leptin on hypothalamic astrocyte morphology and synaptic protein levels. Endocrinology 152, 1809–1818, doi: 10.1210/en.2010-1252 (2011). 21343257 PMC3860256

[b6] Hsuchou H. . Obesity induces functional astrocytic leptin receptors in hypothalamus. Brain 132, 889–902, doi: 10.1093/brain/awp029 (2009). 19293246 PMC2668946

[b7] Cai D. & Liu T. Inflammatory cause of metabolic syndrome via brain stress and NF-kappaB. Aging (Albany NY) 4, 98–115 (2012). 22328600 10.18632/aging.100431 PMC3314172

[b8] Milanski M. . Saturated fatty acids produce an inflammatory response predominantly through the activation of TLR4 signaling in hypothalamus: implications for the pathogenesis of obesity. J Neurosci 29, 359–370, doi: 10.1523/JNEUROSCI.2760-08.2009 (2009). 19144836 PMC6664935

[b9] Kim J. G. . Leptin signaling in astrocytes regulates hypothalamic neuronal circuits and feeding. Nat Neurosci 17, 908–910, doi: 10.1038/nn.3725 (2014). 24880214 PMC4113214

[b10] Garcia-Caceres C. . The opposing effects of ghrelin on hypothalamic and systemic inflammatory processes are modulated by its acylation status and food intake in male rats. Endocrinology 155, 2868–2880, doi: 10.1210/en.2014-1074 (2014). 24848869

[b11] Muller T. D. . Ghrelin. Mol Metab 4, 437–460, doi: 10.1016/j.molmet.2015.03.005 (2015). 26042199 PMC4443295

[b12] Pinto S. . Rapid rewiring of arcuate nucleus feeding circuits by leptin. Science 304, 110–115, doi: 10.1126/science.1089459 (2004). 15064421

[b13] Briggs D. I. & Andrews Z. B. A recent update on the role of ghrelin in glucose homeostasis. Curr Diabetes Rev 7, 201–207 (2011). 21539509 10.2174/157339911795843140

[b14] Leloup C., Orosco M., Serradas P., Nicolaidis S. & Penicaud L. Specific inhibition of GLUT2 in arcuate nucleus by antisense oligonucleotides suppresses nervous control of insulin secretion. Brain Res Mol Brain Res 57, 275–280 (1998). 9675426 10.1016/s0169-328x(98)00097-7

[b15] Bady I. . Evidence from glut2-null mice that glucose is a critical physiological regulator of feeding. Diabetes 55, 988–995 (2006). 16567520 10.2337/diabetes.55.04.06.db05-1386

[b16] Parton L. E. . Glucose sensing by POMC neurons regulates glucose homeostasis and is impaired in obesity. Nature 449, 228–232, doi: 10.1038/nature06098 (2007). 17728716

[b17] Rothstein J. D. . Knockout of glutamate transporters reveals a major role for astroglial transport in excitotoxicity and clearance of glutamate. Neuron 16, 675–686 (1996). 8785064 10.1016/s0896-6273(00)80086-0

[b18] Delgado-Rubin A., Chowen J. A., Argente J. & Frago L. M. Growth hormone-releasing peptide 6 protection of hypothalamic neurons from glutamate excitotoxicity is caspase independent and not mediated by insulin-like growth factor I. Eur J Neurosci 29, 2115–2124, doi: 10.1111/j.1460-9568.2009.06770.x (2009). 19490089

[b19] Lee S., Kim Y., Li E. & Park S. Ghrelin protects spinal cord motoneurons against chronic glutamate excitotoxicity by inhibiting microglial activation. Korean J Physiol Pharmacol 16, 43–48, doi: 10.4196/kjpp.2012.16.1.43 (2012). 22416219 PMC3298825

[b20] Elmquist J. K., Swanson J. J., Sakaguchi D. S., Ross L. R. & Jacobson C. D. Developmental distribution of GFAP and vimentin in the Brazilian opossum brain. J Comp Neurol 344, 283–296, doi: 10.1002/cne.903440209 (1994). 8077462

[b21] Vannucci S. J., Maher F. & Simpson I. A. Glucose transporter proteins in brain: delivery of glucose to neurons and glia. Glia 21, 2–21 (1997). 9298843 10.1002/(sici)1098-1136(199709)21:1<2::aid-glia2>3.0.co;2-c

[b22] De Giorgis V. & Veggiotti P. GLUT1 deficiency syndrome 2013: current state of the art. Seizure 22, 803–811, doi: 10.1016/j.seizure.2013.07.003 (2013). 23890838

[b23] Regan M. R. . Variations in promoter activity reveal a differential expression and physiology of glutamate transporters by glia in the developing and mature CNS. J Neurosci 27, 6607–6619, doi: 10.1523/JNEUROSCI.0790-07.2007 (2007). 17581948 PMC6672708

[b24] Martinez-Hernandez A., Bell K. P. & Norenberg M. D. Glutamine synthetase: glial localization in brain. Science 195, 1356–1358 (1977). 14400 10.1126/science.14400

[b25] Arrieta-Cruz I., Su Y., Knight C. M., Lam T. K. & Gutierrez-Juarez R. Evidence for a role of proline and hypothalamic astrocytes in the regulation of glucose metabolism in rats. Diabetes 62, 1152–1158, doi: 10.2337/db12-0228 (2013). 23274895 PMC3609585

[b26] Rafiki A., Boulland J. L., Halestrap A. P., Ottersen O. P. & Bergersen L. Highly differential expression of the monocarboxylate transporters MCT2 and MCT4 in the developing rat brain. Neuroscience 122, 677–688 (2003). 14622911 10.1016/j.neuroscience.2003.08.040

[b27] Dunn-Meynell A. A., Routh V. H., Kang L., Gaspers L. & Levin B. E. Glucokinase is the likely mediator of glucosensing in both glucose-excited and glucose-inhibited central neurons. Diabetes 51, 2056–2065 (2002). 12086933 10.2337/diabetes.51.7.2056

[b28] Stanley S. . Profiling of Glucose-Sensing Neurons Reveals that GHRH Neurons Are Activated by Hypoglycemia. Cell Metab 18, 596–607, doi: 10.1016/j.cmet.2013.09.002 (2013). 24093682

[b29] Chaudhry F. A. . Glutamine uptake by neurons: interaction of protons with system a transporters. J Neurosci 22, 62–72 (2002). 11756489 10.1523/JNEUROSCI.22-01-00062.2002 PMC6757603

[b30] Pellerin L. & Magistretti P. J. Excitatory amino acids stimulate aerobic glycolysis in astrocytes via an activation of the Na+/K+ATPase. Dev Neurosci 18, 336–342 (1996). 8940604 10.1159/000111426

[b31] Brown A. M. . Astrocyte glycogen metabolism is required for neural activity during aglycemia or intense stimulation in mouse white matter. J Neurosci Res 79, 74–80, doi: 10.1002/jnr.20335 (2005). 15578727

[b32] Reinhart P. H., Pfeiffer B., Spengler S. & Hamprecht B. Purification of glycogen phosphorylase from bovine brain and immunocytochemical examination of rat glial primary cultures using monoclonal antibodies raised against this enzyme. J Neurochem 54, 1474–1483 (1990). 1691273 10.1111/j.1471-4159.1990.tb01194.x

[b33] Muhic M., Vardjan N., Chowdhury H. H., Zorec R. & Kreft M. Insulin and Insulin-like Growth Factor 1 (IGF-1) Modulate Cytoplasmic Glucose and Glycogen Levels but Not Glucose Transport across the Membrane in Astrocytes. J Biol Chem 290, 11167–11176, doi: 10.1074/jbc.M114.629063 (2015). 25792745 PMC4409273

[b34] Chowen J. A., Argente J. & Horvath T. L. Uncovering novel roles of nonneuronal cells in body weight homeostasis and obesity. Endocrinology 154, 3001–3007, doi: 10.1210/en.2013-1303 (2013). 23798599 PMC3749483

[b35] Yang L., Qi Y. & Yang Y. Astrocytes control food intake by inhibiting AGRP neuron activity via adenosine A1 receptors. Cell Rep 11, 798–807, doi: 10.1016/j.celrep.2015.04.002 (2015). 25921535

[b36] Levin B. E., Magnan C., Dunn-Meynell A. & Le Foll C. Metabolic sensing and the brain: who, what, where, and how? Endocrinology 152, 2552–2557, doi: 10.1210/en.2011-0194 (2011). 21521751 PMC3192421

[b37] Morgello S., Uson R. R., Schwartz E. J. & Haber R. S. The human blood-brain barrier glucose transporter (GLUT1) is a glucose transporter of gray matter astrocytes. Glia 14, 43–54, doi: 10.1002/glia.440140107 (1995). 7615345

[b38] Simpson I. A. . The facilitative glucose transporter GLUT3: 20 years of distinction. Am J Physiol Endocrinol Metab 295, E242–253, doi: 10.1152/ajpendo.90388.2008 (2008). 18577699 PMC2519757

[b39] Sakata I. . Glucose-mediated control of ghrelin release from primary cultures of gastric mucosal cells. Am J Physiol Endocrinol Metab 302, E1300–1310, doi: 10.1152/ajpendo.00041.2012 (2012). 22414807 PMC3361986

[b40] Vavaiya K. V., Paranjape S. A. & Briski K. P. Testicular regulation of neuronal glucose and monocarboxylate transporter gene expression profiles in CNS metabolic sensing sites during acute and recurrent insulin-induced hypoglycemia. J Mol Neurosci 31, 37–46 (2007). 17416968 10.1007/BF02686116

[b41] Dienel G. A. Brain lactate metabolism: the discoveries and the controversies. J Cereb Blood Flow Metab 32, 1107–1138, doi: 10.1038/jcbfm.2011.175 (2012). 22186669 PMC3390802

[b42] Marty N. . Regulation of glucagon secretion by glucose transporter type 2 (glut2) and astrocyte-dependent glucose sensors. J Clin Invest 115, 3545–3553, doi: 10.1172/JCI26309 (2005). 16322792 PMC1297256

[b43] Stolarczyk E. . Detection of extracellular glucose by GLUT2 contributes to hypothalamic control of food intake. Am J Physiol Endocrinol Metab 298, E1078–1087, doi: 10.1152/ajpendo.00737.2009 (2010). 20179244

[b44] Thorens B. GLUT2, glucose sensing and glucose homeostasis. Diabetologia 58, 221–232, doi: 10.1007/s00125-014-3451-1 (2015). 25421524

[b45] Gahete M. D. . Ghrelin gene products, receptors, and GOAT enzyme: biological and pathophysiological insight. J Endocrinol 220, R1–24, doi: 10.1530/JOE-13-0391 (2014). 24194510

[b46] Cummings D. E. Ghrelin and the short- and long-term regulation of appetite and body weight. Physiol Behav 89, 71–84, doi: 10.1016/j.physbeh.2006.05.022 (2006). 16859720

[b47] Dailey M. J., Stingl K. C. & Moran T. H. Disassociation between preprandial gut peptide release and food-anticipatory activity. Endocrinology 153, 132–142, doi: 10.1210/en.2011-1464 (2012). 22128024 PMC3249668

[b48] Bulgarelli I. . Desacyl-ghrelin and synthetic GH-secretagogues modulate the production of inflammatory cytokines in mouse microglia cells stimulated by beta-amyloid fibrils. J Neurosci Res 87, 2718–2727, doi: 10.1002/jnr.22088 (2009). 19382238

[b49] Lee J. Y., Oh T. H. & Yune T. Y. Ghrelin inhibits hydrogen peroxide-induced apoptotic cell death of oligodendrocytes via ERK and p38MAPK signaling. Endocrinology 152, 2377–2386, doi: 10.1210/en.2011-0090 (2011). 21467197

[b50] Haam J., Halmos K. C., Di S. & Tasker J. G. Nutritional state-dependent ghrelin activation of vasopressin neurons via retrograde trans-neuronal-glial stimulation of excitatory GABA circuits. J Neurosci 34, 6201–6213, doi: 10.1523/JNEUROSCI.3178-13.2014 (2014). 24790191 PMC4004809

[b51] Langlet F. Tanycytes: a gateway to the metabolic hypothalamus. J Neuroendocrinol 26, 753–760, doi: 10.1111/jne.12191 (2014). 25131689

[b52] Haan N. . Fgf10-expressing tanycytes add new neurons to the appetite/energy-balance regulating centers of the postnatal and adult hypothalamus. J Neurosci 33, 6170–6180, doi: 10.1523/JNEUROSCI.2437-12.2013 (2013). 23554498 PMC3736310

[b53] Collden G. . Neonatal overnutrition causes early alterations in the central response to peripheral ghrelin. Mol Metab 4, 15–24, doi: 10.1016/j.molmet.2014.10.003 (2015). 25685686 PMC4314535

[b54] Fernandez G. . Des-acyl Ghrelin directly targets the arcuate nucleus in a ghrelin-receptor independent manner and impairs the orexigenic effect of ghrelin. J Neuroendocrinol doi: 10.1111/jne.12349 (2015). 26661382

[b55] Balland E. . Hypothalamic tanycytes are an ERK-gated conduit for leptin into the brain. Cell Metab 19, 293–301, doi: 10.1016/j.cmet.2013.12.015 (2014). 24506870 PMC3936883

[b56] Bolborea M. . Melatonin controls photoperiodic changes in tanycyte vimentin and neural cell adhesion molecule expression in the Djungarian hamster (Phodopus sungorus). Endocrinology 152, 3871–3883, doi: 10.1210/en.2011-1039 (2011). 21846800

[b57] Avola R., Di Tullio M. A., Fisichella A., Tayebati S. K. & Tomassoni D. Glial fibrillary acidic protein and vimentin expression is regulated by glucocorticoids and neurotrophic factors in primary rat astroglial cultures. Clin Exp Hypertens 26, 323–333 (2004). 15195687 10.1081/ceh-120034137

[b58] Paul S., Gharami K., Das S. & Sarkar P. K. Thyroid hormone-induced maturation of astrocytes is associated with the expression of new variants of vimentin and their phosphorylation. J Neurochem 73, 1964–1972 (1999). 10537054

[b59] Harada T. . Functions of the two glutamate transporters GLAST and GLT-1 in the retina. Proc Natl Acad Sci USA 95, 4663–4666 (1998). 9539795 10.1073/pnas.95.8.4663 PMC22547

[b60] Yang Y., Atasoy D., Su H. H. & Sternson S. M. Hunger states switch a flip-flop memory circuit via a synaptic AMPK-dependent positive feedback loop. Cell 146, 992–1003, doi: 10.1016/j.cell.2011.07.039 (2011). 21925320 PMC3209501

[b61] Simpson I. A., Carruthers A. & Vannucci S. J. Supply and demand in cerebral energy metabolism: the role of nutrient transporters. J Cereb Blood Flow Metab 27, 1766–1791, doi: 10.1038/sj.jcbfm.9600521 (2007). 17579656 PMC2094104

[b62] McKenna M. C., Sonnewald U., Huang X., Stevenson J. & Zielke H. R. Exogenous glutamate concentration regulates the metabolic fate of glutamate in astrocytes. J Neurochem 66, 386–393 (1996). 8522979 10.1046/j.1471-4159.1996.66010386.x

[b63] Erecinska M. . Glucose and synaptosomal glutamate metabolism: studies with [15N]glutamate. J Neurochem 51, 892–902 (1988). 2900879 10.1111/j.1471-4159.1988.tb01826.x

[b64] Violante I. R. . Cerebral activation by fasting induces lactate accumulation in the hypothalamus. Magn Reson Med 62, 279–283, doi: 10.1002/mrm.22010 (2009). 19526502

[b65] Wierda K. D. & Sorensen J. B. Innervation by a GABAergic neuron depresses spontaneous release in glutamatergic neurons and unveils the clamping phenotype of synaptotagmin-1. J Neurosci 34, 2100–2110, doi: 10.1523/JNEUROSCI.3934-13.2014 (2014). 24501351 PMC6608537

[b66] Egecioglu E. . Ghrelin increases intake of rewarding food in rodents. Addict Biol 15, 304–311, doi: 10.1111/j.1369-1600.2010.00216.x (2010). 20477752 PMC2901520

[b67] Komori T. . Regulation of ghrelin signaling by a leptin-induced gene, negative regulatory element-binding protein, in the hypothalamic neurons. J Biol Chem 285, 37884–37894, doi: 10.1074/jbc.M110.148973 (2010). 20876580 PMC2988391

